# Testcrosses are an efficient strategy for identifying *cis*-regulatory variation: Bayesian analysis of allele-specific expression (BayesASE)

**DOI:** 10.1093/g3journal/jkab096

**Published:** 2021-03-27

**Authors:** Brecca R Miller, Alison M Morse, Jacqueline E Borgert, Zihao Liu, Kelsey Sinclair, Gavin Gamble, Fei Zou, Jeremy R B Newman, Luis G León-Novelo, Fabio Marroni, Lauren M McIntyre

**Affiliations:** 1 Genetics Institute, University of Florida, Gainesville, FL 32608, USA; 2 NYU Langone Health, New York University, New York, NY 10013, USA; 3 Department of Molecular Genetics and Microbiology, University of Florida, Gainesville, FL 32608, USA; 4 Department of Biostatistics, University of North Carolina at Chapel Hill, Chapel Hill, NC 27515, USA; 5 Department of Genetics, University of North Carolina at Chapel Hill, Chapel Hill, NC 27515, USA; 6 Department of Pathology, University of Florida, Gainesville, FL 32608 USA; 7 Department of Biostatistics and Data Science, University of Texas Health Science Center at Houston-University of Texas School of Public Health, Houston, TX 7703, USA; 8 Department of Agricultural, Food, Environmental and Animal Sciences, University of Udine, Udine, 33100, Italy

**Keywords:** allelic imbalance, allele specific expression, Bayesian analysis, *cis*-regulatory variation

## Abstract

Allelic imbalance (AI) occurs when alleles in a diploid individual are differentially expressed and indicates *cis* acting regulatory variation. What is the distribution of allelic effects in a natural population? Are all alleles the same? Are all alleles distinct? The approach described applies to any technology generating allele-specific sequence counts, for example for chromatin accessibility and can be applied generally including to comparisons between tissues or environments for the same genotype. Tests of allelic effect are generally performed by crossing individuals and comparing expression between alleles directly in the F1. However, a crossing scheme that compares alleles pairwise is a prohibitive cost for more than a handful of alleles as the number of crosses is at least (**n^2^-n)/2** where **n** is the number of alleles. We show here that a testcross design followed by a hypothesis test of AI between testcrosses can be used to infer differences between nontester alleles, allowing **n** alleles to be compared with **n** crosses. Using a mouse data set where both testcrosses and direct comparisons have been performed, we show that the predicted differences between nontester alleles are validated at levels of over 90% when a parent-of-origin effect is present and of 60%−80% overall. Power considerations for a testcross, are similar to those in a reciprocal cross. In all applications, the testing for AI involves several complex bioinformatics steps. BayesASE is a complete bioinformatics pipeline that incorporates state-of-the-art error reduction techniques and a flexible Bayesian approach to estimating AI and formally comparing levels of AI between conditions. The modular structure of BayesASE has been packaged in Galaxy, made available in Nextflow and as a collection of scripts for the SLURM workload manager on github (https://github.com/McIntyre-Lab/BayesASE).

## Introduction

Allele-specific expression (ASE) is the amount of mRNA each allele transcribes. Allelic imbalance (AI) indicates a difference in the level of expression of transcripts derived from the two alleles of a diploid individual or among alleles in a polyploidy (Wittkopp *et al.* 2004; Boatwright *et al.* 2018). AI is a result of genetic variation in regulation, both in *cis* (*e.g.*, promoters, enhancers, and other noncoding sequences), and in *trans* (transcription factors). The interpretation of *cis* and *trans* effects depends upon the experimental design deployed (McIntyre *et al.* 2006; Graze *et al.* 2009, 2012, 2014; Fear *et al.* 2016). Testing for regulatory variation that affects expression in *cis* is conceptually straightforward and involves the comparison of the expression profiles of two alleles with the null hypothesis that the expression profiles are equal. If AI is observed, there is direct evidence of *cis* differences between alleles (Wittkopp *et al.* 2004). There are also some potential *cis* by *trans* interactions captured in this comparison (Wittkopp *et al.* 2004; Graze *et al.* 2014). Comparisons of AI across different physiological or environmental conditions is of increasing interest (von Korff *et al.* 2009; Tung *et al.* 2011; Cubillos *et al.* 2014; Buil *et al.* 2015; Chen *et al.* 2015; Pinter *et al.* 2015; Fear *et al.* 2016; Moyerbrailean *et al.* 2016; Knowles *et al.* 2017). Formally testing for differences in AI between conditions reveals environmental effects of variation in *cis* regulation (León-Novelo *et al.* 2018) and can also be used to identify parent of origin effects between reciprocal genotypes (Zou *et al.* 2014).

**Figure 1:**
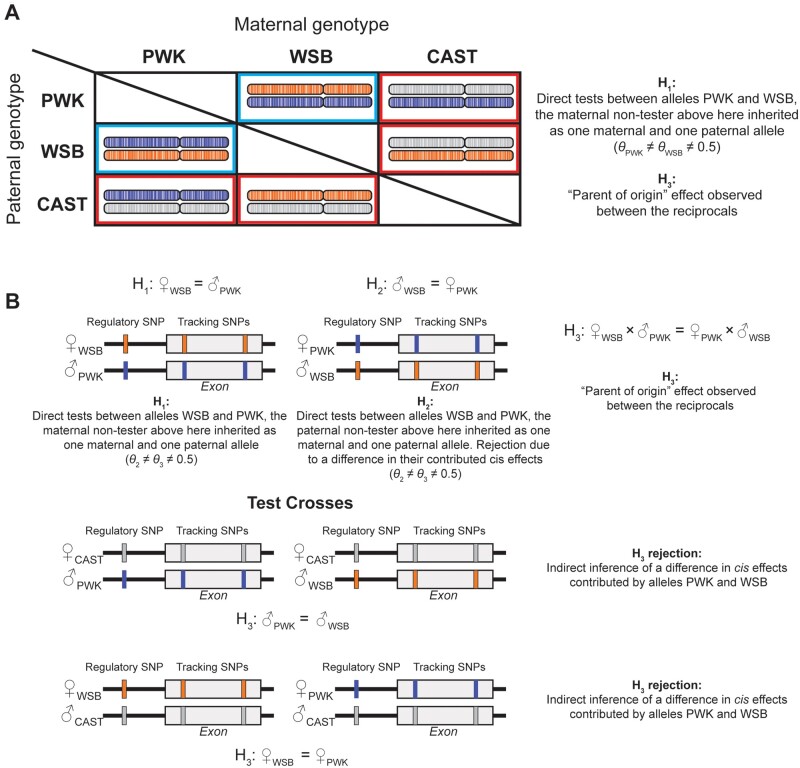
Schematic representation of the crosses used in this study. (A) Maternal genotypes as columns (PWK, WSB, and CAST) and paternal genotypes in rows (PWK, WSB, and CAST). We focus here on the comparison of PWK *vs* WSB; the approach is valid for all the possible comparisons. The test crosses are outlined with red boxes and the direct crosses with blue boxes. H_1_ is a test within any single cross of the null for allelic balance. H_3_ represents a test of the null hypothesis that the AI is the same between two crosses. (B) Alleles PWK and WSB, can be compared directly with a test of H_1_ in either of the two reciprocal crosses in the blue boxes in (A). These tests can be labeled as H_1_ and H_2_ with the label of 1 or 2 assigned arbitrarily. The test of the (H_3_) is then a test of whether the AI is the same between the two reciprocal crosses. A test of H_3_ for the two crosses in the red box can also be conducted, here the test of H_3_ is an indirect inference for difference in *cis* effects contributed by PWK and WSB alleles. Note that the comparison of PWK *vs* WSB in the testcross compares the two alleles inherited from the same parent, while the direct cross is confounded by the parent of origin.

The direct assessment of AI among **n** alleles would require at least (**n^2^-n)/2** crosses. This quickly becomes a very large number; for example, directly testing differences between 10 alleles would require 45 crosses. Theoretically, it is possible to obtain this information by performing **n** crosses, between one tester inbred line and several nontester inbred lines to obtain **n** F1 carrying each the same tester allele and different “line” alleles (Figure 1A, red boxes). Testcrosses (or a reference design) are crosses in which two (or more) nontester alleles are each crossed to a common tester allele. If the nontester alleles do not differ from the tester, then all three alleles (the two nontester and the tester) are similar in their effect. If one of the tester/nontester F1 combinations shows AI but the other does not, this implies that the expression of one of the nontester allele differs from the other nontester/tester. What if both alleles differ from the tester? Does this imply these two alleles are similar? A formal test of whether the AI in these two crosses is equal can be used to identify nontester alleles with divergent *cis* effects. This is an innovative way of estimating allelic effects in a population and predicting alleles likely to differ in *cis*.

There are many bioinformatics steps needed to get to the point where the expression can be compared across conditions; this causes difficulties in reproducibility and has the potential to discourage researchers without extensive bioinformatics capabilities. Differential mapping of the two alleles on the common reference (Degner *et al.* 2009) has led to a demonstration that strain-specific reference improves the estimation of AI and lowers type I error (Skelly *et al.* 2011; Turro *et al.* 2011; Graze *et al.* 2012; Satya *et al.* 2012; León-Novelo *et al.* 2014; Munger *et al.* 2014; Fear *et al.* 2016). This step requires mapping to strain specific references and comparing alignment outputs. Although it should be noted that even with strain-specific mapping, residual bias can persist due to undiscovered structural variants or aspects of sequences that interfere with the mapping algorithms. When DNA can be used as a control, all of these biases are accounted for Wittkopp *et al.* (2004), Graze *et al.* (2009, 2012) but some residual bias may persist for example when gene families are considered. Bias due to sequence similarity across the genome and/or mapping algorithm can be estimated using simulation (Degner *et al.* 2009; Stevenson *et al.* 2013; León-Novelo *et al.* 2014) these estimates can be accounted for in the model reducing type I error (Graze *et al.* 2012). Other bioinformatic challenges include tracking overall expression and nonspecific read mapping, as they both affect the power for detection of AI (Fear *et al.* 2016; León-Novelo *et al.* 2018).

Here, we present BayesASE, a series of clearly elucidated bioinformatic steps modularized and well-documented in a robust high-performing computing (HPC) pipeline. BayesASE requires only reads in FASTQ file format, a reference genome, genotype specific VCF file for variant calling, and a series of design files as input. The tools within the pipeline are modular and provide a clear and reproducible analysis workflow. The template provided makes for transparent substitution and easy execution. Workflow templates have been constructed for SLURM schedulers, for Nextflow (Di Tommaso *et al.* 2017), and for Galaxy (Goecks *et al.* 2010). Galaxy BayesASE consists of individual tools, associated workflows and a detailed conda package environment and associated PyPi package for integration into local Galaxy installs or servers. Individual BayesASE tools are also available for installation from the Galaxy ToolShed. Galaxy is a well-supported open-source environment and training is available here (https://galaxyproject.github.io/training-material/).

While this study focuses on the use of testcrosses for comparison, BayesASE is entirely general. The first design file specifies individual crosses and the corresponding genomes. Any crossing design can be accommodated and the pipeline will then automatically assemble the relevant genomes and count alleles mapping to each parental genome. Furthermore, the unit for which allele-specific reads are to be counted is completely general as the process expects a labeled BED file. This is used throughout the process to identify results for each row of the user-provided BED file. Pairwise comparisons are specified in a separate design file and the only limitation is the requirement that the identifiers on the genomic regions match. For example, two tissues/environments from the same genotype, two sexes, reciprocal crosses, and the testcrosses presented here can all be analyzed with the same process. Our Bayesian model allowing simultaneous estimation of AI in two conditions and direct estimation of the difference in AI between conditions (León-Novelo *et al.* 2018), has been recoded in Stan (Carpenter *et al.* 2017) making the code more robust. Although conceived for measuring AI in expression, our model is suitable for testing allele imbalance in other genomic data, such as assays for chromatin accessibility. The modular development also allows alternate approaches to be included in a straightforward manner. A custom model for AI in a particular circumstance can easily be deployed (Zou *et al.* 2014) instead of the model included with the package while using modules in BayesASE to count allele-specific and nonspecific reads.

## Materials and methods

### BayesASE modules

**Figure 2:**
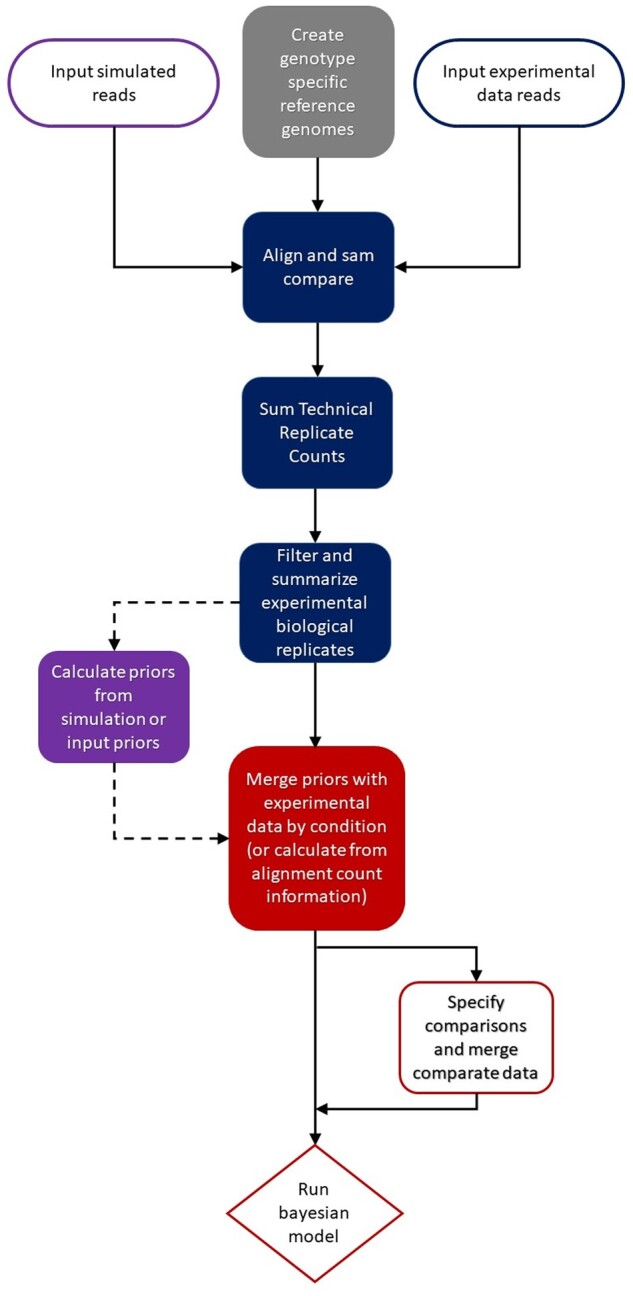
Flowchart of the Bayesian analysis of allele imbalance process for testing for allele imbalance.

BayesASE consists of four main modules: *Genotype Specific References*, *Alignment and SAM Compare*, *Prior Calculation*, and *Bayesian Model* (Figure 2). Flexibility, generality and process checking are present in each of the four modules that make up the BayesASE pipeline. Workflows for each module are coded according to SLURM workflow manager, Nextflow and Galaxy specifications, and all scripts are available on Github (https://github.com/McIntyre-Lab/BayesASE). The Galaxy package BayesASE has a detailed User Guide (Supplementary File S1) that describes the structure of the workflow and all input and output requirements for each of the individual scripts. The code is also available as a PyPI package, a bioconda package and in the Galaxy Toolshed (see Data Availability Section).

The *Genotype Specific References* module (Supplementary Figure S1) requires as input the reference genome as FASTA file, and genotype specific SNP variants as VCF files, and returns as output a set of genotype specific references obtained incorporating SNP variants into the reference genome. Genotype specific reference reduce mapping bias that occurs when a common reference is used (Skelly *et al.* 2011; Turro *et al.* 2011; Graze *et al.* 2012; Satya *et al.* 2012; León-Novelo *et al.* 2014; Munger *et al.* 2014; Fear *et al.* 2016). Input VCF files can be generated using the GATK (DePristo *et al.* 2011), and index input genotype VCF files. BWA index (Li and Durbin 2010) is used to create index files needed for downstream alignment.

The *Alignment and SAM Compare* module (Supplementary Figure S2) quantifies alignment counts for each input file for each of the two genotype specific genomes of the parents of the F1, compares the alignment files in SAM format, and outputs count tables of reads aligned to each parental genome. In this module, input experimental reads for each F1 sample are aligned to each of their updated parental reference genomes with BWA-MEM (Li 2013). Alignment output counts are compared and reads are designated as mapping to one of the parental genomes (or to both when reads mapping equally well to the two genomes). Technical replicates are summed together, and coverage metrics are calculated. The data are then flagged for low coverage (user defined).

The *Bayesian model* requires an estimate of a prior to specify the probability that a read generated from the gene in genome 1 maps better to genome 1 than to genome 2 or is mapping equally well on both genomes for each condition. In this implementation, the prior is specified for each gene/cross and each condition separately allowing for maximum flexibility. For each gene/cross/condition, *q1* is the prior probability that a read originating from genome 1 maps better to genome 1 and *q2* is the prior probability that a read originated from genome 2 maps better to genome 2, with the following constraint: 0 < *q1, q2 *<* *1. The *Prior Calculation* module (Supplementary Figure S3) estimates priors from DNA data, the RNA data, or simulated data. This module receives count tables for read counts for both informative (mapping to a particular parent) and uninformative reads as input and uses them to estimate a prior probability distribution for each given feature. Priors can also derive independently from this tool and supplied directly to the model. Priors provide information on differences in mapping on the two genomes in absence of AI (Graze *et al.* 2012; Fear *et al.* 2016; León-Novelo *et al.* 2018).

The *Bayesian Model* module (Supplementary Figure S4) requires as input a design file to identify comparisons to be performed, alignment count tables and priors, either from the previous module or as direct input. The Bayesian model itself is the STAN (Carpenter *et al.* 2017) implementation of a previously published model for the detection of AI in one or between multiple conditions (León-Novelo *et al.* 2018) with an extension allowing the priors to be independently specified between conditions. Model output files include values for estimates of levels of AI and their 95% central credible intervals. Output also includes the Bayesian measure of evidence against allelic balance (De Bragança Pereira and Stern 1999; Thulin 2014), defined as the smallest number *ev* such that the 1-*ev* central credible interval for estimate of AI does not contain the null value indicating allelic balance. Values of *ev* can be used in a decision theory context to make decisions about rejecting the null hypothesis.

### Workflow deployment platforms and protocol

To facilitate its use, BayesASE is available for SLURM schedulers, as workflows on the Nextflow platform, and as a Galaxy Tool. There is an example set of sbatch scripts for the SLURM implementation on GitHub (https://github.com/McIntyre-Lab/BayesASE). Source code is available under the MIT license. BayesASE has a modular structure; flowcharts of the individual modules outline the logic (Supplementary Figures S1–S4) and detailed information about input/output is described in the Galaxy User Guide available on GitHub and as Supplemental File S1. Names are consistent with the flow charts and standard naming conventions for sbatch and Nextflow are used.

The most direct option that is provided for users is through several templates for sbatch scripts; they can be modified as needed with the input and output file names and submitted to a SLURM scheduler. SLURM is an open-source cluster management and job scheduling system (https://slurm.schedmd.com/); while our work has been focused on SLURM scheduler, our scripts can be used as templates for deployment using a different scheduler. Nextflow (Di Tommaso *et al.* 2017) is a portable, parallelizable and reproducible framework available in the cluster environment. It defines data workflows that can be executed on diverse portable batch system schedulers such as SGE, SLURM, or Cloud platforms. Nextflow pipelines consist of a configuration file and a series of processes that define the major steps in the pipeline. Processes are independently executed from one another and the platform supports a variety of languages such as Bash, Python, R, and so on. Processes are connected through input or output channels, allowing data to be passed through the pipeline. Nextflow also provides an automatic caching mechanism for identifying and skipping successfully completed tasks and using previously cached results for downstream tasks. Each BasyesASE module has been coded in Nextflow and is available on GitHub (https://github.com/McIntyre-Lab/BayesASE). Galaxy is an open-source project designed to be deployed in a web browser, providing a user-friendly platform that can be configured to run on global servers in large universities or on the local individual machines (Goecks *et al.* 2010). Reproducibility in Galaxy is accomplished via histories and the creation of workflows that can be shared among collaborators. The BayesASE modules are developed for use with Galaxy, and have been deposited in the Galaxy Toolshed. Details about how to deploy these tools are in the User Guide.

### Testcrosses can be used to efficiently estimate *cis* effects

To demonstrate that the testcross can be used to predict allelic differences between nontester alleles we analyzed a publicly available data set of male and female F1 *Mus musculus* brain RNA-seq from three different inbred mouse strains, CAST/EiJ, PWK/PhJ, and WSB/EiJ (Zou *et al.* 2014; Crowley *et al.* 2015). The authors provided two alternative notations for the inbred strains: CAST/EiJ = F, PWK/PhJ = G, and WSB/EiJ = H, or alternatively CAST/EiJ = CAST, PWK/PhJ = PWK, and WSB/EiJ= WSB. We denote each F1 sample by its maternal strain × paternal strain. For example, a CAST × WSB mouse is an offspring of a CAST/EiJ female that is mated with a WSB/EiJ male. This same F1 sample can be denoted as FH (short for F × H) in which an F male is mated with an H female. This shorter notation is used to label some tests/figures and the model results. For the original study, the main hypothesis of interest was a test of the null AI_FG_ =AI_GF_, AI_GH_ =AI_HG_, AI_FH_ =AI_HF_, *i.e.* the absence of a parent-of-origin effect. We repeated the analysis with BayesASE, using the same read counts as the original work, and male and female offspring of each cross were analyzed separately as a series of pairwise analyses using the Bayesian model (León-Novelo *et al.* 2018) and not jointly (Zou *et al.* 2014).

The option of performing pairwise analysis of AI is a novel feature of our Bayesian model (León-Novelo *et al.* 2018), in which we introduced the possibility of analyzing AI in two different conditions or genotypes and at the same time testing the hypothesis of difference in the levels of AI between the two conditions. We indicate with H_1_ the hypothesis of allelic balance in condition 1, with H_2_ the hypothesis of allelic balance in condition 2, and with H_3_ the hypothesis of no difference between the levels of AI in conditions 1 and 2 (Figure 1).

Our main interest was to assess whether the theoretical prediction that the testcross can be used to compare different nontester alleles. In the mouse data, the offspring of the crosses CAST × PWK and CAST × WSB, represent a testcross of the PWK and WSB alleles with CAST as the tester allele. Using the flexible design in BayesASE, we estimated AI and tested whether the AI was significant between alleles CAST and PWK in the offspring of CAST × PWK (H_1_ in Figure 1; red boxes), alleles CAST and WSB in the offspring of CAST × WSB (H_2_ in Figure 1, red boxes), and the difference in AI between the two offspring (H_3_ in Figure 1, red boxes). The null hypothesis H_3_ is that the expression of PWK in CAST × PWK is the same as the expression of WSB in CAST × WSB. Because CAST is common to both crosses (and the maternal allele, in this case), testing H_3_ is a test between PWK and WSB. We also compared the test crosses PWK × CAST and WSB × CAST in this case CAST is still the tester allele but here it is inherited paternally. The other two testcross combinations were tested in the same manner. Male and female offspring were evaluated separately. We focus only on the autosomal effects and do not consider the X, Y, or mitochondrial loci.

To verify the predictions made in the testcross, the reciprocal crosses were used (Figure 1, blue boxes). For example, to verify differences in the PWK and WSB alleles predicted by the CAST × PWK and CAST × WSB cross we examined the PWK × WSB and WSB × PWK crosses. Because the alleles in the testcrosses are compared with the same parental inheritance and the alleles in the direct test have different parental inheritance, we do not expect that 100% of the predictions will be realized. In addition, the *trans* environments are different between the testcrosses, with *trans* effects of the tester allele shared but differing in the nontester allele. The Spearman coefficient of correlation was used to compare the estimates for AI estimated from BayesASE with the estimates of AI used in the original work (Zou *et al.* 2014; Crowley *et al.* 2015) for the 95 imprinted genes and all data were analyzed using the same counts from the original analysis.

## Data availability

All scripts, together with a test data set and a readme file are available at https://github.com/McIntyre-Lab/BayesASE, and as a PyPi repository (https://pypi.org/project/BayesASE/), and a bioconda package (https://anaconda.org/bioconda/bayesase). A detailed User Guide providing step by step instructions for the each module and detailed instructions for the Galaxy interface is included as Supplementary File S1. Supplemental Material available at figshare: https://doi.org/10.25387/g3.14174291. All tools are deposited in the Galaxy ToolShed for download and installation (https://testtoolshed.g2.bx.psu.edu/repository?repository_id=ef69fe5507b8d8c7&changeset_revision=8b2027117ce5). The mouse data are available from prior publications (Zou et al. 2014, Crowley et al. 2015).

## Results and discussion

Estimates of allelic effect obtained with BayesASE were concordant with the published estimates of AI from the 95 imprinted genes using the model described by (Zou *et al.* 2014; Crowley *et al.* 2015). Supplementary Figure S5 shows, for the reciprocal CAST × PWK and PWK × CAST crosses, the estimated AI as the ratio of the paternal to the maternal allele. All the correlation coefficients are greater than 0.96, even for reciprocal crosses. The correlation for the same cross in the present study and published results is always 1. Using the analysis in BayesASE, we report the *cis* effects as estimated by tests of AI for each cross. The estimates range from 5.39 to 11.74% of genes showing AI for each cross (Table 1) and an overall estimate of 3629 (26%) loci of the 14, 058 that were tested for all crosses in both sexes. As these data have been analyzed and reported on elsewhere (Zou *et al.* 2014; Crowley *et al.* 2015), we will focus only on the testcross results.

**Table 1:** Percentage of genes showing AI in different crosses

	AI (%)	
Cross	Females	Males
PWK WSB	7.38	8.03
WSB PWK	8.34	7.87
PWK CAST	7.06	6.44
CAST PWK	7.23	5.38
CAST WSB	8.38	10.7
WSB CAST	8.72	11.23

**Figure 3:**
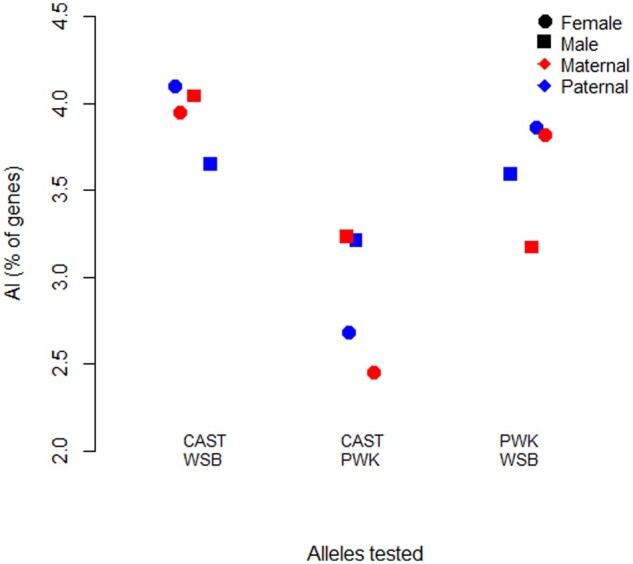
Percentage of genes showing AI, tested via the testcross approach. The *x*-axis represents the two alleles being compared. CAST and WSB are compared by testing H3 in the two crosses CAST × PWK and WSB × PWK (to test Maternal contribution, red) or PWK × CAST and PWK × WSB (to test Paternal contribution, blue). Results for female offspring are shown as circles, and for male offspring are shown as squares.

The testcross predicts ∼1200 loci with *cis* effects in PWK × WSB, ∼900 loci with *cis* effects in CAST × PWK and ∼1000 in CAST × WSB, accounting for 2–5% of the tested genes (Figure 3). The frequency of *cis* effects was similar regardless of the parent of origin. Using BayesASE, we directly tested the null that the AI between the offspring sexes was equal for each of the 6 F1 crosses. The detection of *cis* effects was similar for male and female offspring with the exception of the CAST × WSB cross where AI was 2x more likely to be identified in male offspring compared to female offspring (*P* < 0.0001 McNemar’s test). An additional complication in the *cis* predictions of direct crosses is the impact of the parental inheritance on the allelic expression. In the testcross the comparison is between the two alleles inherited from the maternal (or paternal) parent, while in the direct cross one allele is inherited maternally and the other is inherited paternally.

More than 70% of *cis* effects predicted by the testcross as differences between nontester alleles (*i.e.*, rejection of the null hypothesis H_3_) are validated in the direct comparison (Table 2) for all comparisons except the CAST × WSB male which has a validation rate of 46%, or approximately 3 times higher than expected based on the frequency of *cis* effects in the direct crosses. The validation rate when parent of origin effects were present was higher. For genes in which the null hypothesis H_3_ was rejected between reciprocal crosses were identified as having parent of origin effect. When limiting the comparison of the testcross to the direct cross to genes where a parent-of-origin effect was present the validation rates were greater than 90% in all cases (Table 2). Of note, the CAST × WSB male offspring have a much lower proportion of reciprocal effects than the other five comparisons.

**Table 2:** Validation rates of testcrosses using testcrosses

Alleles compared	Sex	*N* total	Validated total	*N* POO	Validated POO
PWK CAST	Female	691	68.89	510	91.96
PWK CAST	Male	776	63.14	533	90.81
PWK WSB	Female	938	72.6	721	92.93
PWK WSB	Male	916	73.58	713	92.85
CAST WSB	Female	879	74.63	699	92.56
CAST WSB	Male	787	46.25	388	92.53

Number of genes showing different levels of expression using testcrosses (*N* total). Percentage of genes showing different levels of expression using testcrosses validated in direct comparison, in total (validated total). Number of genes showing different levels of expression using testcrosses and having parent of origin effect in the reciprocal crosses (*N* POO). Percentage of genes showing different levels of expression using testcrosses and having parent of origin effect, validated in direct comparison (validated POO).

These results indicate that a testcross design is an efficient way of identifying *cis* effects. Instead of a joint pairwise **n^2^-n** experiment, it is possible to plan an experiment of size **n**, where **n** is the number of alleles tested in one sex or paternal/maternal effects. There are some subtleties worth considering when thinking about *cis* effects in organisms with a heterogametic sex system. The testcross will compare nontester alleles inherited from either the maternal or paternal parent. The direct comparison, in contrast, will always compare the maternally inherited allele to the paternally inherited allele. When there are no interactions between the heterogametic sex chromosome or cytoplasmic factors and the autosomes, this should result in the same loci being identified. However, *trans* acting factors from the heterogametic sex chromosome and cytoplasmic factors complicates the interpretation of the direct comparison, particularly in males.

A more technical potential explanation is the potential difference in power for these two approaches. effect of expression on power. In the comparison between testcrosses there is potential for reduced power, compared to the direct cross (León-Novelo *et al.* 2018) as the test of H3 between crosses may have lower power than the test of H1. The number of allele-specific reads affects power. When testing the H_3_ hypothesis for difference of AI between two conditions, power is affected by the number of allele-specific reads from the experiment with the lowest coverage (León-Novelo *et al.* 2018). In addition, overall gene expression, if not accounted for, may affect power (Fear *et al.* 2016). We note that in the model presented here this factor has been effectively accounted for, minimizing this issue (León-Novelo *et al.* 2018).

The mouse data show that the use of testcrosses to compare nontester alleles identifies loci that are validated by the direct tests in the vast majority of cases. Loci identified in direct crosses are identified in test crosses but there are many more loci identified in the direct cross. This may be a false negative result for the testcross approach, due to lower power and/or it may be due to differences in differences in the number of allele-specific read counts for one of the crosses that lowers the power for H3. However, it may well be that there is a *cis-trans* interaction between the heterogametic sex chromosome and the autosomal genes, and/or a parent of origin effect in the direct cross.

The parent of origin effect can be tested by comparing AI between reciprocal crosses. We can examine whether this effect explains some of the differences in identification of *cis* effects in the testcross compared to identified in the direct cross. For 60–75% of the loci with a parent of origin effect there was evidence for a *cis* effect in the direct cross as well, with the exception of the CAST × WSB male where 46% of the parent of origin effects had corresponding *cis* effects. This is logical, as at least one of the reciprocal crosses must have a relatively large estimate of AI in order to detect the parent of origin effect. The difference in the CAST × WSB male may not be as surprising as at first glance. This is a cross between genetically distant lines (Zou *et al.* 2014) and may reflect divergence in gene expression between the sexes in these incipient species due to sex antagonism. When focusing on only those genes with a parent of origin effect and a *cis* effect in the direct cross, the validation rate for the testcross is comparable, and large (>90%) for all crosses.

The testcross approach is a useful strategy to maximize allele comparison while minimizing sequencing efforts. Testcrosses will not detect either parent of origin or *cis-trans* interactions since the comparison between alleles is from a shared maternal/paternal inheritance. The reciprocal effect is large in these data indicating that either parent of origin and/or *cis-trans* interactions are important in these data, consistent with the original data analysis (Crowley *et al.* 2015). Other work has also implicated *trans*-acting factors from the × influencing ASE on the autosomes (Graze *et al.* 2014). The efficacy of the testcross is clear from these data, also clear is the presence of *cis-by-trans* effects from the X, mitochondrial or Y chromosome influencing expression variation in autosomal genes in the mouse.

BayesASE has been used here to test for *cis* effects in individual crosses, differences in *cis* effects between males and females of a single cross, differences in *cis* between testcrosses, and parent of origin/cis-trans interactions in reciprocal crosses. The BayesASE framework lays out each step to testing AI transparently, and enables researchers to perform analysis using their preferred approach (galaxy, Nextflow, or SBATCH queuing). We provide a set of well-documented python scripts organized into modules and available with examples as SLURM bash jobs, using Nextflow or Galaxy. BayesASE its designed in a modular fashion. Users can rely on the whole pipeline of analysis, select a specific step, or replace a specific step. The addition of usable accessible code should make these more complex models and bioinformatics steps more accessible to the community.

The Bayesian method to analyze ASE presented here (León-Novelo *et al.* 2018) is completely general. In this study, we present an application to a testcross however, the full pipeline and analysis can also be applied to comparing the same genotypes in different environmental conditions (Fear *et al.* 2016). The statistical test presented of difference between conditions is complemented by a test of allele imbalance within each condition. While there are a number of such tests that have been developed, this particular approach addressed the impacts of bias, and expression level variation. When ignored, both of these effects can increase type I error (León-Novelo *et al.* 2014, 2018; Fear *et al.* 2016). The model itself is general and can be applied to any technology producing allele-specific read counts including ChIP-seq and Hi-C. By providing transparent code for the complete pipeline in three formats we hope to facilitate the use of more sophisticated statistical approaches by the broad scientific community.
